# Supplementary material for: Deep learning for obstructive sleep apnea diagnosis based on single channel oximetry
Source: Nat Commun. 2023 Aug 12;14:4881. doi: 10.1038/s41467-023-40604-3 (PMC10423260; doi:10.1038/s41467-023-40604-3)
Supplement: Supplementary file 1 — Supplementary Information [file 41467_2023_40604_MOESM1_ESM.pdf]

Supplementary Figures

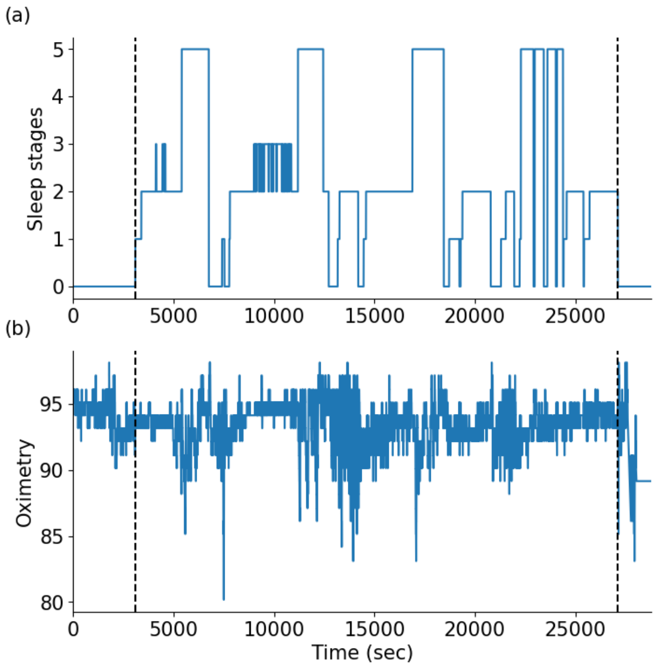

Figure S1: On panel (a), the sleep stages. On panel (b), the oximetry time series. Sleep stage of 0 means that the patient is awake. The vertical dotted lines mark the beginning and end of the truncated oximetry signal.

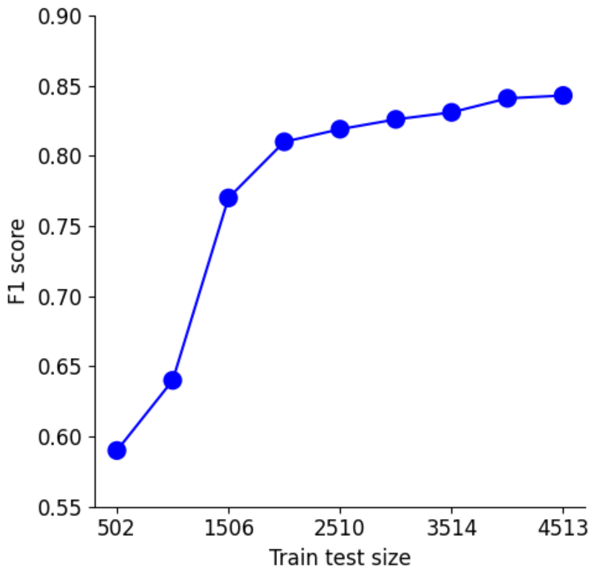

Figure S2: F1 score of OxiNet on SHHS1-test as a function of the train set size. The curve shows a monotonous increase as the training set size increases.

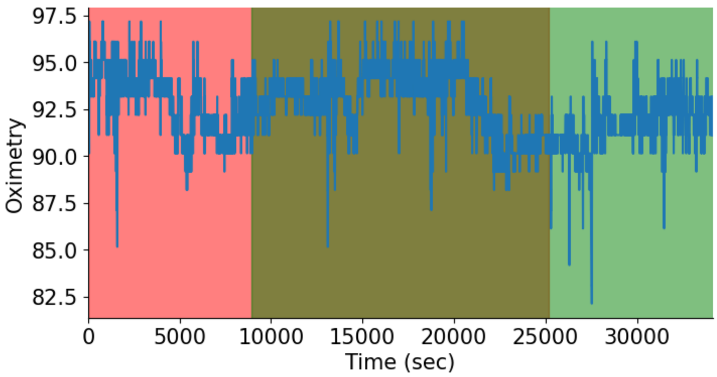

Figure S3: Show the moving window technique, for data augmentation. The red and green rectangles are 2 different samples for the model, taken from the same signal. The brown rectangle in the center is because the red and green colors overlap.

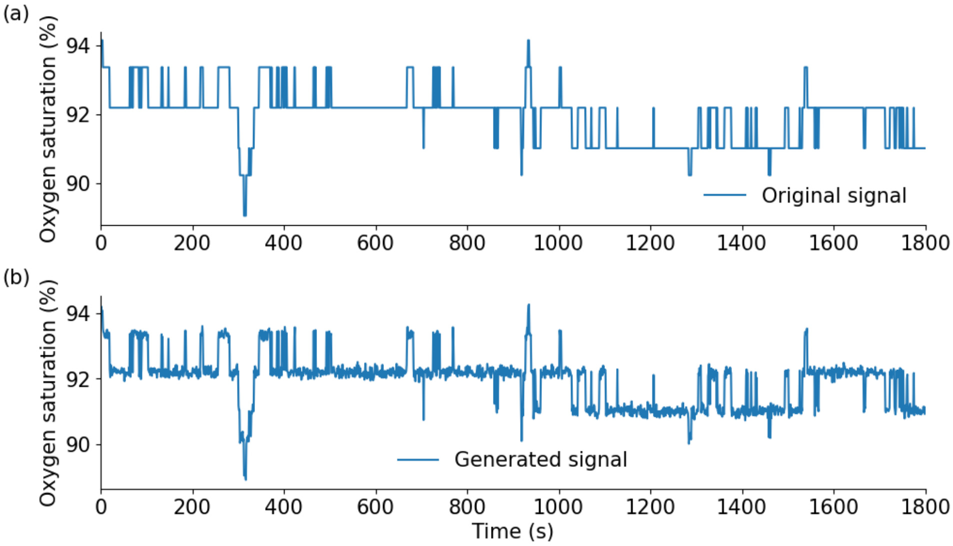

Figure S4: On panel (a), the original signal. On panel (b), the generated signal with Jitter augmentation. A subset of the whole signal is presented, to appreciate the different patterns existing in the signal.

Supplementary Tables

Hyper-parameter	Value
L_{window}	3600 seconds
n_B	3
n_L	3
n_{DB}	2
n_C	8
$K_{dilated}$	5
$rate_{dilation}$	4
N_{CNN}	256
n_{CRNN}	4
k_{CRNN}	9
n_{LSTM}	4
N_{CRNN}	256
N_{META}	4
$n_{classifier}$	3
$d_{classifier}$	0.3

Table S1: Different hyper-parameters of OxiNet found in the bayesian search.

Database	R^2	ICC	PPV_M	Se_M	$F_{1,M}$	κ
SHHS1 ($n = 562$)	0.86	0.93	0.76	0.73	0.74	0.64
SHHS2 ($n = 605$)	0.87	0.92	0.77	0.74	0.74	0.65
UHV ($n = 357$)	0.85	0.93	0.68	0.70	0.74	0.61
CFS ($n = 586$)	0.65	0.75	0.60	0.58	0.60	0.44
MROS ($n = 3753$)	0.76	0.81	0.67	0.62	0.65	0.54
MESA ($n = 2002$)	0.66	0.75	0.72	0.64	0.65	0.59

Table S2: Results on the test set of the external databases, with the CatBoost model trained on SHHS1 database using oximetry biomarkers (i.e. feature engineering) as input.

Ethnicity	Number	OxiNet	
		ICC	$F_{1,M}$
All test set	2,002	0.94	0.75
Hispanic	481	0.71	0.72
Black and African American	547	0.90	0.71
white	729	0.95	0.78
Chinese American	245	0.95	0.77

Table S3: F1 score for different ethnicities of MESA database.

Database	Name	Normal	Mild	Moderate	Severe	$p - value$
SHHS1	Number	1648	1879	1161	699	
	Gender (m)	70%	52%	37%	31%	$p = 9.7e^{-7}$
	Age	60.00 ± 17.0	66.00 ± 16.00	66.00 ± 15.75	66.00 ± 17.00	$p = 8.4e^{-16}$
	BMI	26.25 ± 5.39	28.30 ± 5.89	29.17 ± 7.03	30.52 ± 7.76	$p = 1.1e^{-6}$
	Smoking status	832 / 214 / 602	827 / 167 / 885	523 / 75 / 563	307 / 51 / 341	$p = 6.7e^{-8}$
SHHS2	Number	571	885	559	412	
	Gender (m)	71%	58%	44%	33%	$p = 2.5e^{-8}$
	Age	64.50 ± 15.0	69.00 ± 15.00	71.00 ± 15.00	71.50 ± 15.00	$p = 8.9e^{-16}$
	BMI	26.20 ± 5.58	28.25 ± 6.00	29.33 ± 6.22	29.87 ± 8.25	$p = 4.2e^{-6}$
	Smoking status	308 / 54 / 209	452 / 62 / 371	281 / 27 / 251	200 / 20 / 192	$p = 6.0e^{-8}$
UHV	Number	28	62	66	200	
	Gender (m)	46%	63%	82%	82%	$p = 4.2e^{-10}$
	Age	46.00 ± 22.0	55.00 ± 16.50	60.00 ± 19.50	58.00 ± 16.00	$p = 1.8e^{-14}$
	BMI	25.50 ± 5.60	25.71 ± 4.94	27.79 ± 4.73	30.39 ± 6.17	$p = 6.1e^{-12}$
	Smoking status	11 / 7 / 10	15 / 17 / 30	13 / 12 / 41	45 / 49 / 106	$p = 0.06$
CFS	Number	264	101	69	69	
	Gender (m)	60%	56%	45%	27%	$p = 5.2e^{-4}$
	Age	41.00 ± 21.0	54.00 ± 19.00	51.00 ± 16.50	52.00 ± 27.00	$p = 2.9e^{-7}$
	BMI	27.00 ± 11.00	35.00 ± 10.00	33.00 ± 10.50	38.00 ± 10.25	$p = 3.8e^{-3}$
	Smoking status	178 / 54 / 32	42 / 37 / 22	30 / 19 / 20	29 / 16 / 24	$p = 4.4e^{-2}$
MROS	Number	3928	200	18	8	
	Gender (m)	100%	100%	100%	100%	–
	Age	78.0 ± 8.0	77.5 ± 7.00	79.0 ± 8.0	80.0 ± 8.0	$p = 3.7e^{-14}$
	BMI	25.00 ± 5.00	26.00 ± 5.00	27.00 ± 4.00	28.00 ± 6.00	$p = 9.4e^{-6}$
	Smoking status	NA	NA	NA	NA	–
MESA	Number	406	638	512	469	
	Sex (m)	30%	40%	50%	65%	$p = 3.4e^{-9}$
	Age	65.00 ± 14.0	68.00 ± 15.00	70.00 ± 14.00	69.00 ± 14.00	$p = 6.4e^{-20}$
	BMI	NA	NA	NA	NA	–
	Smoking status	NA	NA	NA	NA	–

Table S4: Descriptive statistics of the clinical data, for the five databases used. The four features are presented: gender, age, BMI, and smoking status. For continuous features, the Median (MED) and interquartile range are presented. For categorical features, the distribution among the categories is presented. Smoking status data are provided for (No smoker / Smoker / Ex-smoker). The p-value of the one-sided Kruskal Wallis test are presented in the table. NA stands for not available.

Database	R^2	ICC	PPV_M	Se_M	$F_{1,M}$	κ
SHHS1 ($n = 562$)	0.92	0.96	0.83	0.87	0.84	0.77
SHHS2 ($n = 605$)	0.93	0.95	0.82	0.85	0.83	0.76
UHV ($n = 357$)	0.9	0.92	0.77	0.79	0.77	0.72
CFS ($n = 586$)	0.83	0.92	0.8	0.78	0.78	0.68
MROS ($n = 3753$)	0.91	0.94	0.78	0.85	0.80	0.74
MESA ($n = 2002$)	0.86	0.94	0.76	0.75	0.75	0.68

Table S5: Results on the test set of the external databases, with OxiNet trained on 90% of SHHS1.

Statistics			
1	AV	Blood oxygen saturation (SpO_2) mean	%
2	MED	SpO_2 median	%
3	Min	SpO_2 min	%
4	SD	SpO_2 standard deviation	%
5	RG	SpO_2 range	%
6	Px	x^{th} percentile SpO_2 value	%
7	Mx	Percentage of the signal at least x% below median oxygen saturation	%
8	ZCx	Number of zero-crossing points at the x% SpO_2 level,	n.u.
9	ΔIx	Delta index	%
Complexity			
10	ApEn	Approximate entropy	n.u.
11	LZ	Lempel–Ziv complexity	n.u.
12	CTM_p	Central tendency measure	n.u.
13	SampEn	Sample entropy	n.u.
14	DFA	Detrended fluctuation analysis	%
Periodicity			
15	$PRSAD_c$	Phase-rectified signal averaging (PRSA) capacity	%
16	$PRSAD_ad$	PRSA amplitude difference	%
17	$PRSAD_os$	PRSA overall slope	%/s
18	$PRSAD_sb$	PRSA slope before the anchor point	$\frac{\%}{s}$
19	$PRSAD_sa$	PRSA slope after the anchor point	$\frac{\%}{s}$
20	AC	Autocorrelation	$\%^2$
21	PSD_{total}	The integral of the power spectral density (PSD) function	%
22	PSD_{band}	The integral of the PSD function within the band $0.0140.033Hz$	%
23	PSD_{ratio}	PSD_{band} with respect to the total integral	n.u.
24	PSD_{peak}	Peak amplitude of the PSD function within the band 0.0140.033 Hz	%

Table S6: List of digital oximetry biomarkers for the categories: general statistics, complexity, and periodicity.

Desaturation			
25	ODI_x	The oxygen desaturation index	event/h
26	DL_{μ}	Mean of desaturations length	s
27	DL_{σ}	Std of desaturations length	s^2
28	$DDmax_{\mu}$	Mean of desaturations depth	%
29	$DDmax_{\sigma}$	Std of desaturation depth	$\%^2$
30	$DD100_{\mu}$	Mean of desaturations depth using 100% as baseline	%
31	$DD100_{\sigma}$	Std of desaturations depth using 100% as baseline	$\%^2$
32	DS_{μ}	Mean of the desaturation slope	$\frac{\%}{s}$
33	DS_{σ}	Std of the desaturation slope	$\frac{\%}{s}^2$
34	$DAmax_{\mu}$	Mean of the desaturation areas using the maximum value	$\% * s$
35	$DAmax_{\sigma}$	Std of desaturation area	$(\% * s)^2$
36	$DA100_{\mu}$	Mean of desaturation area under 100% as baseline	$\% * s$
37	$DA100_{\sigma}$	Std of desaturation area under 100% as baseline	$(\% * s)^2$
38	TD_{μ}	Mean of time between two consecutive events	s
39	TD_{σ}	Std of time between two consecutive events	s^2
Hypoxic Burden			
40	POD_x	Time of oxygen desaturation event, normalized by TRT	s
41	AOD_{max}	The area under the oxygen desaturation event curve, using the maximum SpO_2 value as baseline and normalized by the TRT	%
42	AOD_{100}	Cumulative area of desaturations under the 100% SpO_2 level as baseline and normalized by the TRT	%
43	CT_x	Cumulative time below the x% oxygen saturation level	%
44	CA_x	Integral of SpO_2 below the x SpO_2 level normalized by the TRT	%

Table S7: List of digital oximetry biomarkers for the categories: desaturation measures and hypoxic burden.

Configuration	ICC	$F_{1,M}$
OxiNet no sleep onset/offset	0.90	0.80
OxiNet no-AUG	0.92	0.81
**OxiNet**	**0.96**	**0.84**

Table S8: Results on SHHS1-test of OxiNet with different configurations. no-AUG: without data augmentation.

	Se	Sp	Acc	PPV	NPV
SHHS1	0.9	0.92	0.95	0.96	0.88
SHHS2	0.88	0.92	0.95	0.96	0.89
UHV	0.8	0.91	0.96	0.97	0.85
CFS	0.85	0.88	0.87	0.84	0.92
MROS	0.78	0.94	0.94	0.94	0.94
MESA	0.83	0.77	0.92	0.97	0.57

Table S9: Different performance measures for OxiNet, when applying a threshold of 5 on the AHI - meaning classifying between no-OSA and OSA. The performance measures are computed on the test set of each database. Se stands for sensitivity, Sp for specificity, Acc for accuracy, PPV for positive predictive value, and NPV for negative predictive value.

	Se	Sp	Acc	PPV	NPV
SHHS1	0.93	0.93	0.93	0.95	0.91
SHHS2	0.92	0.92	0.92	0.89	0.96
UHV	0.92	0.93	0.94	0.96	0.9
CFS	0.93	0.91	0.93	0.84	0.98
MROS	0.94	0.94	0.94	0.93	0.95
MESA	0.91	0.9	0.91	0.95	0.86

Table S10: Different performance measures for OxiNet, when applying a threshold of 15 on the AHI - meaning classifying between no-OSA / mild OSA and moderate/severe OSA. The performance measures are computed on the test set of each database. Se stands for sensitivity, Sp for specificity, Acc for accuracy, PPV for positive predictive value, and NPV for negative predictive value.

	OxiNet				
	Se	Sp	Acc	PPV	NPV
SHHS1	0.88	0.96	0.96	0.97	0.96
SHHS2	0.9	0.95	0.96	0.95	0.96
UHV	0.91	0.91	0.91	0.96	0.86
CFS	0.95	0.96	0.98	0.94	0.98
MROS	0.9	0.93	0.94	0.92	0.94
MESA	0.9	0.94	0.93	0.95	0.93

Table S11: Different performance measures for OxiNet, when applying a threshold of 30 on the AHI - meaning classifying between severe OSA and others. The performance measures are computed on the test set of each database. Se stands for sensitivity, Sp for specificity, Acc for accuracy, PPV for positive predictive value, and NPV for negative predictive value.

Supplementary Notes

POBM features

The POBM features have been extracted from the toolbox developed by Levy et al.[35] OBMs definitions are presented in Table S6 and S7.

The desaturations and hypoxic burden features have been extracted both with relative and hard threshold.[13] For the relative threshold, we use 3 and 5. For the hard threshold, we use 83, 85, and 90. The PRSA features have been extracted with windows of lengths 10 and 20.

Losses

Additional performance measures

The coefficient of determination (R^2) was computed:

$$R^2 = 1 - \frac{\sum_{i=1}^{n}(y_i - \widehat{y_i})^2}{\sum_{i=1}^{n}(y_i - \bar{y})^2} \quad (7)$$

where $\bar{y} = \frac{1}{n}\sum_{i=1}^{n} y_i$, $\widehat{y_i}$ is the predicted AHI of the model, and y_i is the actual AHI (the target). The kappa measure was computed as well:

$$\kappa = \frac{p_0 - p_e}{1 - p_e} \quad (8)$$

p_0 is the relative observed agreement among ratters whereas p_e is the hypothetical probability of chance agreement.

Added value

In addition, we observed that data augmentation significantly improved ($p < 0.05$) OxiNet performance (Table S8). Processing the oximetry time series from sleep onset to sleep offset (in contrast to taking a window systematically starting after 30-min of the start recording time) led to a significant improvement in OxiNet performance with the SHHS1-test set ($ICC = 0.96$ versus $ICC = 0.90$ and $F_{1,M} = 0.84$ versus $F_{1,M} = 0.80$; Table S8). These results emphasize the need to distinguish between sleep and wake to reach high performance with OxiNet. When considering a single pulse oximeter-based diagnosis system, this can be achieved using the photoplethysmography (PPG) signal recorded by the pulse oximeter.[26]
